# Marine Sponges and Bacteria as Challenging Sources of Enzyme Inhibitors for Pharmacological Applications

**DOI:** 10.3390/md15060173

**Published:** 2017-06-12

**Authors:** Nadia Ruocco, Susan Costantini, Flora Palumbo, Maria Costantini

**Affiliations:** 1 Department of Biology and Evolution of Marine Organisms, Stazione Zoologica Anton Dohrn, Villa Comunale, 80121 Napoli, Italy; nadia.ruocco@szn.it; 2 Department of Biology, University of Naples Federico II, Complesso Universitario di Monte Sant’Angelo, Via Cinthia, 80126 Napoli, Italy; 3 Bio-Organic Chemistry Unit, Institute of Biomolecular Chemistry-CNR, Via Campi Flegrei 34, Pozzuoli, 80078 Naples, Italy; 4 Unità di Farmacologia Sperimentale, Istituto Nazionale Tumori “Fondazione G. Pascale”, IRCCS, 80131 Napoli, Italy; susancostantini77@gmail.com; 5 Department of Integrative Marine Ecology, Stazione Zoologica Anton Dohrn, Villa Comunale, 80121 Napoli, Italy; mcosta@szn.it; 6 Institute of Biosciences and BioResources, CNR, 80131 Napoli, Italy

**Keywords:** enzyme inhibitors, sponges, bacteria

## Abstract

Enzymes play key roles in different cellular processes, for example, in signal transduction, cell differentiation and proliferation, metabolic processes, DNA damage repair, apoptosis, and response to stress. A deregulation of enzymes has been considered one of the first causes of several diseases, including cancers. In the last several years, enzyme inhibitors, being good candidates as drugs in the pathogenic processes, have received an increasing amount of attention for their potential application in pharmacology. The marine environment is considered a challenging source of enzyme inhibitors for pharmacological applications. In this review, we report on secondary metabolites with enzyme inhibitory activity, focusing our attention on marine sponges and bacteria as promising sources. In the case of sponges, we only reported the kinase inhibitors, because this class was the most representative isolated so far from these marine organisms.

## 1. An Introduction to Enzyme Inhibitors in Marine Environments

An important challenge of the last several decades has been the search for active compounds from natural sources, about 50% of which have pharmacological applications [1,2].

Among natural compounds, enzyme inhibitors have been considered useful tools mainly for their biotechnological potential in pharmacology [3] and agriculture [4]. In particular, protease inhibitors represent important examples of enzyme inhibitors, able to inactivate target proteases in the presence of human diseases (as for example in high blood pressure, arthritis, muscular dystrophy, pancreatitis, thrombosis, different cancers, as well as AIDS [5,6]). In the case of carbohydrate-dependent diseases, such as diabetes, obesity and hyperlipemia, amylase inhibitors represent useful tools for controlling them [7,8]. Enzyme inhibitors have been also isolated from different terrestrial organisms, including microorganisms (mainly actinomycetes; [9]), even if they are able to produce structurally identical inhibitors [10]. Differently, in the marine organisms occurs considerably different characteristics of enzyme inhibitors in comparison with the terrestrial ones [11].

In fact, the oceans (covering 70% of the earth) represent a widely unexplored and promising source of new biologically active natural compounds, also linked to the highest biodiversity of the marine environment in comparison with the terrestrial one [12,13,14,15,16]. Concerning the marine environment, it is important to consider that sessile marine invertebrates, such as sponges, cnidaria, and ascidians, constitute the largest amount of biomass of the marine macrofauna. They are able to store several secondary metabolites for their ecological success in different marine habitats so as to counterattack predation and pathogenic organisms [17,18,19,20]. These chemical defenses of sessile marine invertebrates not only have ecological roles but also exhibit several different biological activities with pharmacological, nutraceutical, and cosmaceutical applications [13,21,22,23].

Marine microorganisms isolated for the first time in the marine environment in the last century are another key source of active natural compounds. In more detail, marine microorganisms are very different from terrestrial ones, for their metabolic and physiological processes, because about 90% of them are Gram-negative psychrophiles that also require high Na^+^ concentration for growth [24,25]. Maeda and Taga [25] isolated a deoxyribonuclease from a marine *Vibrio* sp. and studied its activity, demonstrating that this enzyme was activated by Mg^2+^ and stabilized by Ca^2+^. Moreover, another marine bacterium has been isolated, able to produce a phosphatase with its activity at a high level of hydrostatic pressure (1000 atm; [26]), suggesting that marine enzymes acted in a different way in comparison to terrestrial ones. Moreover, many enzymes have significant roles in the maintenance of homeostasis and diseases are the results of the breakdown of homeostasis. In fact, there is a very strong correlation between the biological functions of several enzymes and diseases. Enzyme inhibitors are molecules that reduce the catalytic activity or the complete blocking of enzymes, thus causing either the complete cell death or modification in the pathways. On this line, some enzyme inhibitors correlated with specific diseases are very important as drugs [27]. For example, the angiotensin-converting enzyme (ACE) inhibitors act as antihypertensive drugs, reducing hypertension. Adenosine deaminase inhibitors alter adenosine and deoxyadenosine levels together with lympocytic growth and function, thus enhancing the chemotherapeutic effects of adenoside analogs in cancer chemotherapy [28].

Taking this into account, this review reports examples of many enzyme inhibitors from marine microorganisms with pharmaceutical significance. In particular, we report on two important sources of enzyme inhibitors from the marine environment: the sponges and the bacteria. In the case of sponges, we will only focus our attention on the kinase inhibitors since this class is the most representative that has so far been isolated from these marine organisms.

## 2. Marine Sponges

Although marine sponges are considered very simple marine organisms, they represent “chemical factories” because they are able to produce a great number of biologically active compounds [29]. There is still an ongoing debate about whether natural products isolated from sponges originated from sponges or from associated bacteria. Several experiments have evidenced that some compounds isolated from sponges are synthesized by their associated microorganisms [15,30]. In the case of enzyme inhibitors, there are also several evidences on their bacterial origin (see below).

**Figure 1:**
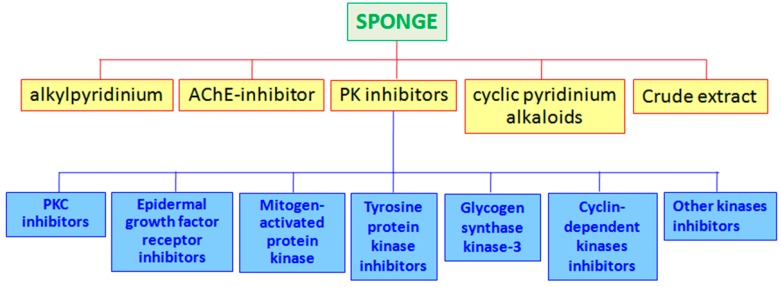
Enzyme inhibitors isolated from marine sponges.

**Figure 2:**
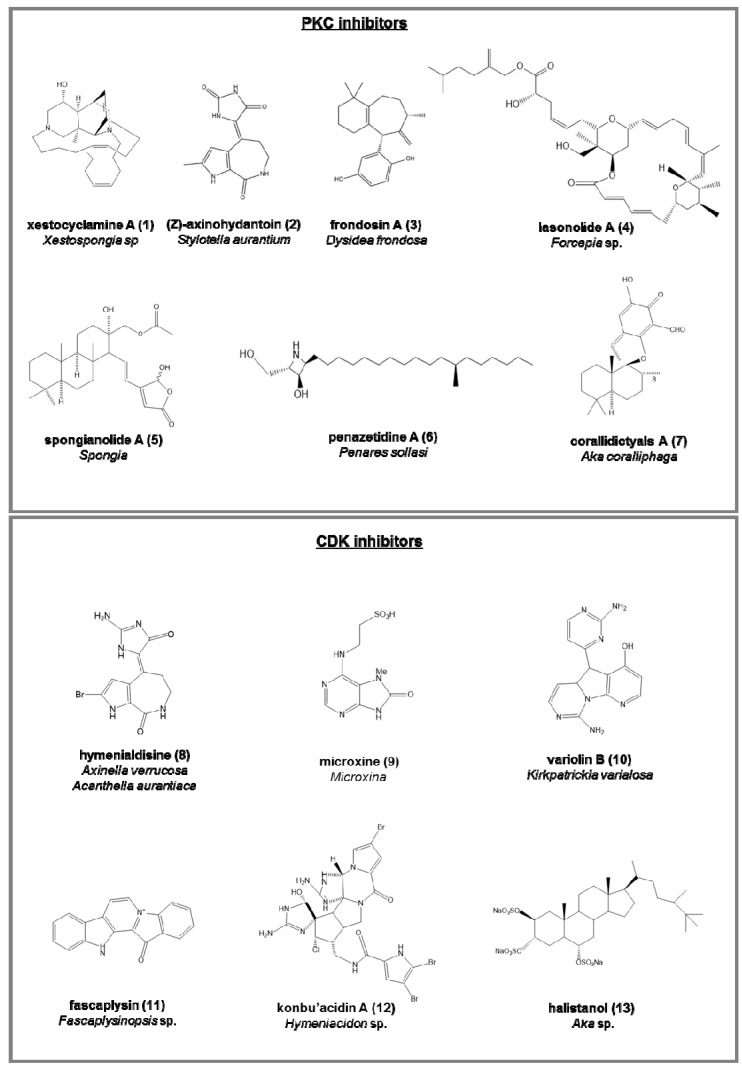
Chemical structures of some natural PKC and CDK inhibitors isolated from sponges, reported as examples.

**Figure 3:**
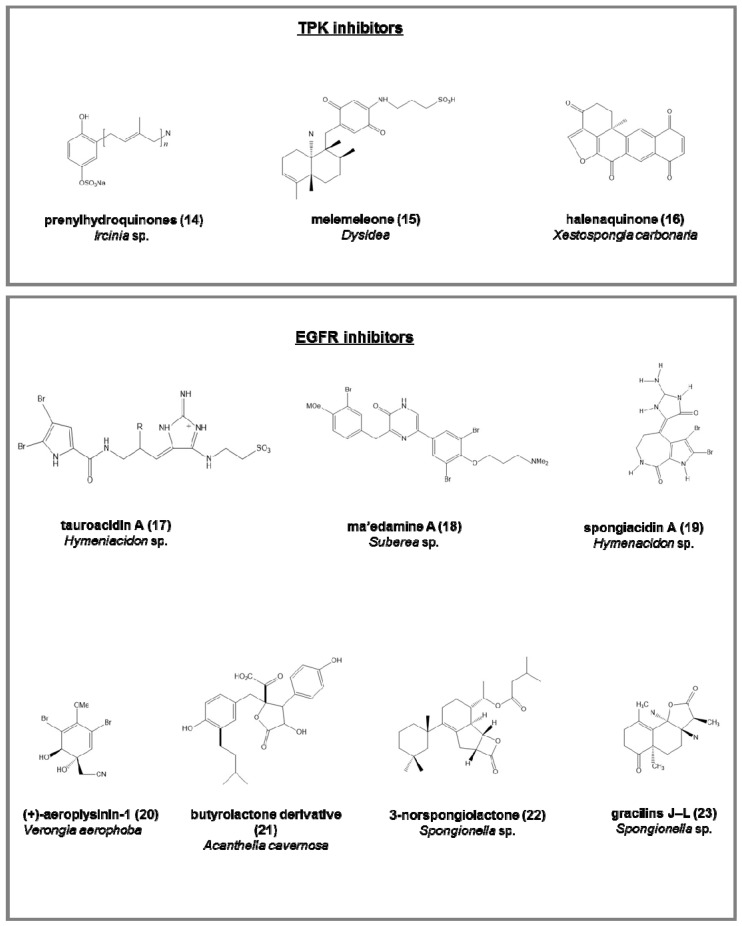
Chemical structure of some natural TPK and EGFR inhibitors isolated from sponges, reported as examples.

**Figure 4:**
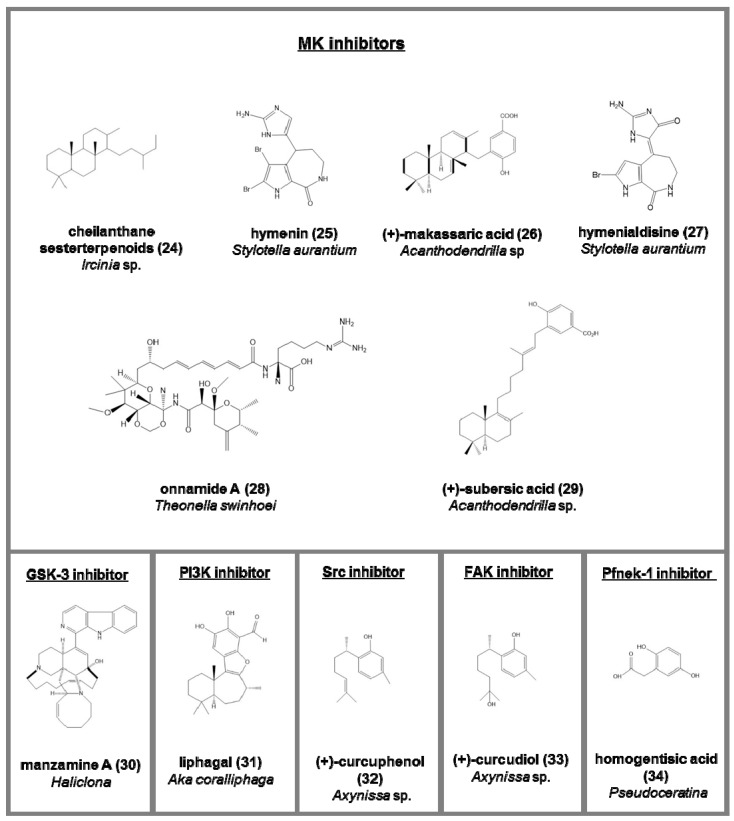
Chemical structure of some natural mitogen-activated protein kinase (MK) and GSK-3 inhibitors and other kinases inhibitors isolated from sponges, reported as examples.

Polyketides, terpenoids, and peptides are the most abundant products isolated from sponges, showing inhibitory activities against many enzymes (Figure 1; see also Figure 2, Figure 3 and Figure 4 for the chemical structures of enzyme inhibitors reported in this paragraph).

Recently, the alkylpyridinium salts have been isolated from sponges, showing potent biological activities mainly as enzyme inhibitors. The marine sponge *Reniera sarai* produce some polymeric 3-alkylpyridinium salts [29]. Cyclostelletamines, cyclic pyridinium alkaloids, from the sponge *Stelletta maxima* [31] are able to inhibit the reaction of methyl quinuclidinyl benzylate with muscarinic acetylcholine receptors. The sponge *Callyspongia fibrosa* is the source of some polymers, which inhibit the epidermal growth factors [32]. Among acetylcholinesterase (AChE) inhibitors, an irreversible inhibitor (the onchidal) has been isolated from the mollusk *Onchidella binneyi* [33], and a pseudozoanthoxanthin-like compound from the coral *Parazoanthus axinellae* [34].

The enzyme adenine phosphoribosyl transferase of *Leishmania tarentolae* (L-APRT) has been inhibited by crude extracts from several marine invertebrates, as for example from the ascidian *Polysyncraton* sp. and from the bryozoan *Bugula* sp. [35]. Concerning the sponges, an inhibitor of L-APRT activity has been found in *Dragmacidon* sp. and *Polymastia* sp. The haplosclerid *Callyspongia* sp. SS97-23 was the source of three meroterpenoids, ilhabelanol, ilhabrene, and isoakaterpin, very potent inhibitors of L-APRT [36]. The marine sponge *Petromica* sp. BA99-103 produced halistanol sulfate, another inhibitor of L-APRT [35].

Marine sponges have been also proven as sources of protein kinase (PK) inhibitors. The enzymes, belonging to the protein kinase family, chemically catalyzed the transfer of a phosphate group to a defined substrate from a high-energy molecule. About 2% of all eukaryotic genes are protein kinases, organized in eight main groups [37]: (1) TK (tyrosine kinase); (2) TKL (tyrosine kinase-like); (3) STE (STE20, STE11, and STE7); (4) CK1 (casein kinase 1); (5) AGC (protein kinase A, protein kinase G, and protein kinase C); (6) CAMK (Ca^2+^/calmodulin-dependent kinases); (7) CMGC (Cdk, MAPK, GSK, Cdk-like); (8) RGC (receptor guanylyl cyclase). Kinases play key roles in different regulatory cellular processes, signal transduction, cell proliferation and differentiation, metabolic processes, apoptosis, and so on [2,38]. Because of the very different roles of the kinases, several diseases have as causes the deregulation of these enzymes. Furthermore, misregulation of various kinases has very often been reported in cancerous cells, so anticancer treatments involve kinases to specifically target cancer cells [39]. Kinase inhibitors represented a good challenge for cancer treatments, considering that to date about 130 kinase inhibitors are in different phases of clinical trials [40]. This is the case, for example, of Imitinib (Gleevec, Novartis), a tyrosine kinase inhibitor used in the prognosis for sufferers of chronic myeloid leukemia, now in the pharmaceutical market [41]. Inhibitors for different PK isolated from marine sponges are reported below (Table 1).

PKC inhibitors (serine/threonine kinases involved in cell differentiation, apoptosis, and inhibition cancer; Figure 2):

- xestocyclamine A (**1**), from *Xestospongia* sp. from Papua New Guinea coast, used for the development of drugs in anticancer therapy [42,43,44];
- (Z)-Axinohydantoin (**2**) and debromo-Z-axinohydantoin, from *Stylotella aurantium* [45];
- five sesquiterpene derivatives, frondosins A–E (frondosin A (**3**) in Figure 1) from *Dysidea frondosa*. Frondosins A–E were inhibitors of interleukin-8, whereas frondosins A and D also have activity against the HIV virus [46];
- BRS1, a lipid from a calcarea sponge [47];
- nakijiquinones A–D, sesquiterpenoid quinones, from a *Spongiidae* [48,49], exerting also inhibitory activity against HER2 kinase and epidermal growth factor receptor (EGFR) [50];
- lasonolide A (**4**), from *Forcepia* sp., used against mouse thymoma cells [51,52,53];
- spongianolides A–E (spongianolide A (**5**) in Figure 2), sesterpenes, from the genus *Spongia* [54];
- penazetidine A (**6**), new azetidine compound from *Penares sollasi*, cytotoxic against human and murine cancer cell lines (A549, HT-29, B16/F10 and P388) [55];
- corallidictyals A (**7**) and B, two diastereomeric spirosesquiterpene aldehydes, from *Aka coralliphaga* [56], selectively inhibiting the α-PKC isoform.

Cyclin-dependent kinases (CDK) inhibitors (serine/threonine kinases, involved in the regulation of the cell cycle; Figure 2):

CDK-1 (mitosis phase)

- hymenialdisine (**8**), from Axinella verrucosa and Acanthella aurantiaca, able to act on NF-kappa B signaling process and indicated as possible pharmaceuticals in treating rheumatoid arthritis, multiple sclerosis, and Alzheimer’s diseases by some recent patents [57];
- microxine (**9**), a purine derivative from genus *Microxina* [58];
- variolin B (**10**), from the Antarctic *Kirkpatrickia varialosa*, able to inhibit the phosphorylation process of histone H1 [59,60].

CDK-4 (G1 phase)

- fascaplysin (**11**), a red pigment isolated from *Fascaplysinopsis* sp. It is a potent inhibitor of angiogenesis, inhibiting the proliferation of endothelial cells through VEGF [61].
- konbu’acidin A (**12**), a bromopyrrole alkaloid from the *Okinawan Hymeniacidon* sp. [62];
- two quinols and halistanol sulfate (**13**), novel sesquiterpene, from *Aka* sp. [63];

Tyrosine protein kinase (TPK) inhibitors (involved in the phosphorylation of tyrosine residues in proliferative diseases; Figure 3):

- the penta-, hexa-, and hepta-prenylhydroquinone 4-sulfates (**14**), from the deep-sea *Ircinia* sp. [64], with cytotoxic activity against HIV-1 integrase enzyme and epidermal carcinoma cell line;
- melemeleone (**15**), a novel sesquiterpene, from two species of Dysidea, with activity against tyrosine kinase pp60V-SRC [65];
- halenaquinone (**16**), halenaquinol, halenaquinol sulfate, and xestoquinone from Xestospongia carbonaria [66].

Epidermal growth factor receptor (EGFR) inhibitors (tyrosine kinases involved mainly in breast cancer; Figure 3):

- tauroacidins A (**17**) and B, bromopyrrole alkaloids from *Hymeniacidon* sp. [67];
- ma’edamine A (**18**), bromotyrosine alkaloid, from *Suberea* sp. [68];
- spongiacidins A (**19**) and B, bromopyrrole alkaloids from *Hymenacidon* sp. [69];
- (+)-aeroplysinin-1 (**20**), from *Verongia aerophoba*, antitumoral on EGFR-dependent human breast cancer cell lines MCF-7 and ZR-75-1 [70];
- butyrolactone derivative (**21**), from *Acanthella cavernosa* [71];
- 3-norspongiolactone (**22**) and gracilins J–L (**23**), bioactive diterpenes from *Spongionella* sp. [72].

Mitogen-activated protein kinase (MK) inhibitors (serine/threonine protein kinases associated to stress; Figure 4):

- cheilanthane sesterterpenoid (**24**), from Ircinia sp. [73] and extracts from *Iotrochota birotulata* and *Spongia barbara* [74];
- hymenin (**25**) and hymenialdisine (**27**), from *Stylotella aurantium* [75];
- a methanol fraction from *Batzella* sp. [76];
- onnamide A (**28**), heterocyclic compounds belonging to pederin family from *Theonella swinhoei*. Its activity is through the stimulation of plasminogen activator inhibitor-1 (PAI-1) considered a drug target against metastasis in human cancer cells [77];
- (+)-makassaric acid (**26**) and (+)-subersic acid (**29**), meroterpenoids from *Acanthodendrilla* sp., acting inflammatory responses and cellular stress processes [78].

Glycogen synthase kinase-3 (GSK-3) inhibitors (a serine/threonine protein kinase, involved in neurodegenerative diseases; Figure 4):

- manzamine A (**30**), an alkaloid from the genus Haliclona [79], agent against the growth of the malaria parasite *Plasmodium berghei* [80].

Other kinases inhibitors (Figure 4)

- liphagal (**31**), a meroterpenoid from *Aka coralliphaga*, with inhibitory activity against PI3K and cytotoxic against human colon and human breast cancer [81];
- (+)-curcuphenol (**32**) (Src protein kinase inhibitor) and (+)-curcudiol (**33**) (focal adhesion kinase, FAK, inhibitor), two bisabolenes type sesquiterpenoids *Axynissa* sp. [82];
- (**34**) homogentisic acid from *Pseudoceratina* against protein kinase of *Plasmodium falciparum* [83];
- hymenialdisine, debromohymenialdisine, and four novel dihydrohymenialdisine derivatives from *Cymbastela cantharella*, able to inhibit the Polo-Like kinase-1 [84].

## 3. Marine Bacteria

**Figure 5:**
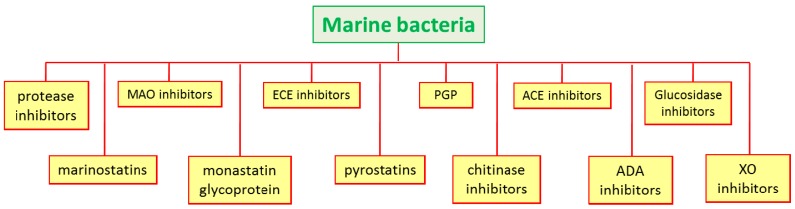
Enzyme inhibitors isolated from marine bacteria.

In the marine environment, several enzyme inhibitors have been isolated from bacteria and actinomycetes for their industrial applications and their use in medicine and agriculture [11] (see Figure 5).

One of the first isolated marine microbial source of enzyme inhibitors were represented by the *Alteromonas* sp., from which serine and cysteine protease inhibitors have been isolated [85]. An example of serine protease inhibitors are represented by the marinostatins C-1 and C-2, used as drugs in pancreatitis pathogenesis thanks to its properties to inhibit α-chymotrypsin (IC_50_ = 1.0–3.2 µM) [86,87].

Inhibitors for different PK isolated from marine bacteria are summarized in Table 2.

**Figure 6:**
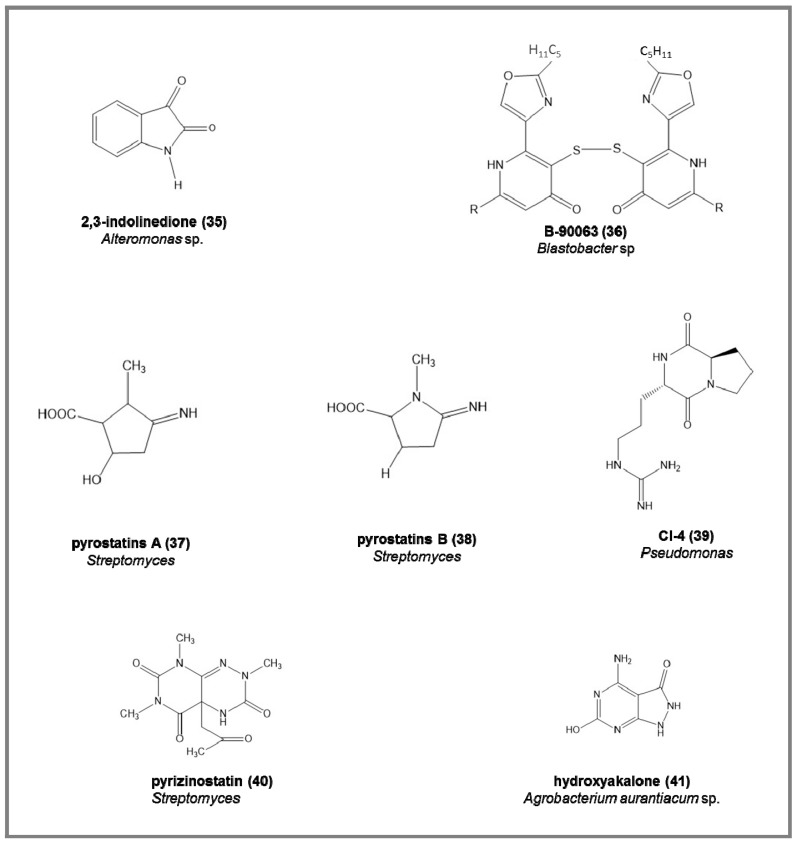
Examples of chemical structure of some enzyme inhibitors isolated from marine bacteria.

Some examples of the chemical structures of enzyme inhibitors from marine bacteria are reported in Figure 6:

- endogenous monoamine oxidase (MAO) inhibitors, the 2,3-indolinedione (**35**), from marine *Alteromonas* sp., increasing the acetylcholine and dopamine in neurotransmission processes (IC_50_ = 9.2 µM) [88].
- monastatin, a glycoprotein from pathogenic fish bacteria such as *Aeromonas hydrophila* and *Vibrio anguillarum* [89]. It has been applied in the production of cooked fish meat gel [90].
- B-90063 (**36**), endothelin-converting enzyme (ECE) inhibitor from *Blastobacter* sp. SANK 71894, (IC_50_ = 1.0–3.2 μM) [91], able to cause blood vessel contraction and for this reason applied in hypertension, cardiovascular, and renal diseases [92].
- pyrostatins A (**37**) and B (**38**), from the *Streptomyces* strain SA-3501, (IC_50_ = 1.0 μM), with inhibitory activity against *N*-acetyl-b-d-glucosaminidase (GluNAc-ase; [93]), applied as drugs in diabetes [94], leukemia [95], and cancer [96].
- pyrizinostatin (**40**), inhibitor of pyroglutamyl peptidase (PGP) from a *Streptomyces* strain (IC_50_ = 21 μM) [97], able to block thyrotropin-releasing and luteinizing hormone-releasing hormones [98].
- CI-4 **(39)** (cyclo(l-Arg-d-Pro; [99,100]), a chitinase inhibitor from *Pseudomonas* sp., a useful source for antifungal and insecticidal agents [101].
- angiotensin-converting enzyme (ACE) inhibitors and adenosine deaminase inhibitors (ADA) [27], from different *Streptomyces* strains. ACE inhibitors were able to reduce hypertension either by the suppression of angiotensin II biosynthesis or by the stimulation of bradykinin breakdown; ADA inhibitors were responsible for the alteration in adenosine and deoxyadenosine levels and in lymphocytic growth and functions, and enhance the effects of chemotherapeutic effects of adenosine analogs. Among 94 *Streptomyces* strains screened, 8 and 4 strains were positive for ACE and ADE inhibitors, respectively [27].
- hydroxyakalone (**41**), an inhibitor of xanthine oxidase (XO), from the fermentation broth of *Agrobacterium* aurantiacum sp. (IC_50_ = 4.6 μM) [102], used as a drug in diseases caused by an accumulation of uric acid [103].

Recently, the actinobacteria have also been considered as producers of enzyme inhibitors. This is the case for 30 strains of marine actinobacteria reported by Ganesan et al. [104], used in treating diabetes.

Marine bacteria associated with the sponge *Jaspis* sp. have been characterized for their production of protease inhibitors [105]. In particular, the associated bacteria *Providencia* sp. showed inhibitory activity against the protease subtilisin, and *Bacillus* sp. had inhibitory effects against the metalloproteinase thermolysin.

## 4. General Conclusions

The increased incidence of severe diseases, including cancer, prompted the scientific research to find new drugs. This also pushed the scientific communities to explore the marine environment for new pharmaceuticals. In fact, the marine environment has a great richness and biodiversity of micro- and macroorganisms, also exhibiting biosynthetic pathways to produce secondary metabolites useful both against predators and active compounds for human health attracting the attention of pharmaceutical industries. A very significant example is represented by several classes of enzyme inhibitors isolated from marine sponges and bacteria, as reported in the review. These enzyme inhibitors show cytotoxic activity against many different cancer cell lines (see Table 1). Marine-derived enzyme inhibitors are considered viable alternatives in pharmaceuticals for replacing synthetic drugs to combat, for example, cancer as well as viral, amtiinflammatory, and neurodegenerative diseases. Nonetheless, further studies will be necessary to investigate the human consumer’s well being. The general significance of the topic of this review is well underlined by the number of EU-funded projects, as well as by Horizon 2020, which are aimed at improving the exploitation of marine organisms for drug discovery.

## Figures and Tables

**Table 1:** Kinase enzyme, enzyme inhibitors names (together with number compounds in the case the chemical structure have been reported in Figure 2, Figure 3, Figure 4 and Figure 5), references, pharmacological applications, and IC_50_ values (in micromolar, μM) from marine sponges: the four less potent enzyme inhibitors were indicated with (*), the five more potent with (**).

Kinase Enzyme	Compound	Reference	Pharmacological Application	IC_50_ (μM)
**PKC**				
	xestocyclamine A (**1**)	[42,43,44]	anticancer	10
	(Z)-Axinohydantoin (**2**)	[45]		9
	frondosin A (**3**)	[46]	HIV virus	1.8
	frondosin B	[46]	HIV virus	4.8
	frondosin C	[46]	HIV virus	20.9
	frondosin D	[46]	HIV virus	26
	frondosin E	[46]	HIV virus	30.6
	nakijiquinones A	[48,49]	anticancer	270 *
	nakijiquinones B	[48,49]	anticancer	200 *
	nakijiquinones C	[48,49]	anticancer	23
	nakijiquinones D	[48,49,50]	anticancer	220 *
	lasonolide A (**4**)	[51,52,53]	thymoma cells	0.03 **
	spongianolides A (**5**) –E	[54]		20–30
	penazetidine A (**6**)	[55]	anticancer	1
	corallidictyals A (**7**) –B	[56]		28
**CDK**				
	hymenialdisine (**8**)	[57]	rheumatoid arthritis	0.02 **
	microxine (**9**)	[58]		13
	variolin B (**10**)	[59,60]	antiviral, anticancer	0.03
	fascaplysin (**11**)	[61]	anticancer, angiogenesis	0.4
	konbu’acidin A (**12**)	[62]	anticancer	20
	halistanol (**13**)	[63]	anticancer	0.013 **
	penta-prenylhydroquinone 4-sulfates (**14**)	[64]	antiviral, anticancer	8
	hexa-prenylhydroquinone 4-sulfates	[64]	antiviral, anticancer	4
	hepta-prenylhydroquinone 4-sulfates	[64]	antiviral, anticancer	8
	melemeleone (**15**)	[65]	anticancer	28
	halenaquinone (**16**)	[66]	anticancer	1.5
**EGFR**				
	tauroacidin A (**17**)	[67]	anticancer	0.001 **
	ma’edamine A (**18**)	[68]	anticancer	11
	spongiacidin A (**19**)	[69]		8.5
	spongiacidin B	[69]		6
	(+)-aeroplysinin-1 (**20**)	[70]	anticancer	0.25–0.5
	butyrolactone derivative (**21**)	[71]	anticancer	22.9
	3-norspongiolactone (**22**)	[72]	anticancer	0.6–15
	gracilins J–L (**23**)	[73]	anticancer	0.6–15
**MK**				
	cheilanthane (**24**)	[74]	anticancer	4
	hymenin (**25**)	[75]		128.8–250 *
	hymenialdisine (**27**)	[76]	antitumor	0.003–0.006 **
	onnamide A (**28**)	[77]	anti-inflammatory	30
	(+)-makassaric acid (**26**)	[78]	anti-inflammatory	20
	(+)-subersic acid (**29**)	[78]	anti-inflammatory	9.6
**GSK-3**				
	manzamine A (**30**)	[79,80]	Alzheimer’s disease	10.2
**Other kinases inhibitors**				
	liphagal (**31**)	[81]	anticancer	0.1
	(+)-curcuphenol (**32**)	[82]		36
	homogentisic acid (**34**)	[83]	antimalarial	1.8

**Table 2:** Enzyme, enzyme inhibitors names (together with number compounds in the case the chemical structure have been reported in Figure 6), references, biotechnological applications, and IC_50_ values (in micromolar, μM) from marine bacteria.

Kinase Enzyme	Compound	Reference	Application	IC_50_ (μM)
**Serine protease**	marinostatins C1–C2	[86,87]	pancreatitis pathogenesis	1.0–3.2
**Monoamine oxidase**	2,3-indolinedone (**35**)	[88]	neurodegenrative diseases	9.2
**Protease**	monostatin	[89,90]	cooked fish meat gel	
**ECE**	B-90063 (**36**)	[92]	hypertension, renal disease	1.0–3.2
**GluNAc-ase**	pyrostatins A–B (**37**,**38**)	[93,94,95,96]	diabetes, leukemia, cancer	1
**PGP**	pyrizinostatin (**40**)	[97,98]	hormone diseases	21
**Chitinase**	CI-4 (**39**)	[99,100,101]	antifungal and insecticidal	
**Xanhine oxidase**	hydroxyakalone (**41**)	[102,103]	uric acid accumulation	4.6
